# Evaluation of the Optimal Manufacturing Protocols and Therapeutic Properties of Mesenchymal Stem/Stromal Cells Derived from Wharton’s Jelly

**DOI:** 10.3390/ijms24010652

**Published:** 2022-12-30

**Authors:** Monika Sypecka, Aleksandra Bzinkowska, Dorota Sulejczak, Filip Dabrowski, Anna Sarnowska

**Affiliations:** 1 Mossakowski Medical Research Institute, Translational Platform for Regenerative Medicine, Polish Academy of Sciences, 02-106 Warsaw, Poland; 2 Department of Experimental Pharmacology, Mossakowski Medical Research Institute, Polish Academy of Sciences, 02-106 Warsaw, Poland; 3 Department of Obstetrics, Perinatology and Neonatology, Center for Medical Postgraduate Education, 01-813 Warsaw, Poland

**Keywords:** Wharton’s jelly, mesenchymal stem/stromal cells, cerebrospinal fluid, stem cell therapy, neural differentiation

## Abstract

Wharton’s jelly (WJ) from the umbilical cord (UC) is a good source of mesenchymal stem/stromal cells (MSCs), which can be isolated and used in therapy. Current knowledge shows that even small changes in the cell environment may result in obtaining a subpopulation of cells with different therapeutic properties. For this reason, the conditions of UC transportation, cell isolation, and cultivation and the banking of cells destined for clinical use should be unified and optimized. In this project, we tried various protocols for cell vs. bioptat isolation, banking, and transport in order to determine the most optimal. The most efficient isolation method of WJ-MSCs was chopping the whole umbilical matrix with a scalpel after vessel and lining membrane removal. The optimal solution for short term cell transportation was a multi-electrolyte fluid without glucose. Considering the use of WJ-MSCs in cell therapies, it was important to investigate the soluble secretome of both WJ bioptats and WJ-MSCs. WJ-MSCs secreted higher levels of cytokines and chemokines than WJ bioptats. WJ-MSCs secreted HGF, CCL2, ICAM-1, BDNF, and VEGF. Since these cells might be used in treating neurodegenerative disorders, we investigated the impact of cerebrospinal fluid (CSF) on WJ-MSCs’ features. In the presence of CSF, the cells expressed consecutive neural markers both at the protein and gene level: nestin, β-III-tubulin, S-100-β, GFAP, and doublecortin. Based on the obtained results, a protocol for manufacturing an advanced-therapy medicinal product was composed.

## 1. Introduction

Stem cells are non-specialized cells with the ability to self-renew, proliferate, and differentiate into different types of cells [1,2]. They have regenerative properties and are not only able to repopulate damaged tissue but, through the secretion of different immunomodulatory factors, can modulate the process of inflammation as well (many of the secreted factors modulate the reparation processes in situ) [3,4,5]. Thanks to these properties, stem cells might be used in therapy concerning diseases that remain resistant to pharmacological treatment, such as Alzheimer’s disease, amyotrophic lateral sclerosis (ALS), multiple sclerosis (MS), and spinal cord injuries [6,7]. Although it is becoming increasingly clear that mesenchymal stem/stromal cells (MSCs) have a weak capability to functionally repopulate injured tissue, their regenerating effect based on secretory properties has been repeatedly proven and has become the basis for therapeutic use in many diseases [8,9]. MSCs can be isolated from different tissues, for example, human umbilical cord, adipose tissue, and bone marrow [10,11,12]. MSCs obtained from different sources usually represent a highly heterogenic population of cells and differ when it comes to clonogenic properties, proliferation potential, soluble secretome, and senescence rate [13,14]. The number of cells isolated from a specific tissue varies between patients and is associated with patients’ health, well-being, age, gender, and individual features and depends on many mechanical factors such as isolation procedure or the conditions of cell transportation. Nowadays, the isolation of MSCs from afterbirth tissues (e.g., Wharton jelly/umbilical matrix) is becoming more popular, as these cells can be stored for a prolonged time and then used in regenerative therapy [11,15,16,17,18]. Wharton’s jelly (WJ) is a connective tissue (made up of numerous collagen fibers, hyaluronic acid, and several sulfated glycosaminoglycans) within the UC, which surrounds the blood vessels, protecting them from intrauterine pressure and ensuring blood flow [18,19,20,21,22]. There are different isolation methods for WJ-MSCs, including a mechanical method and an enzymatic method. While WJ-MSCs, WJ bioptats, and whole umbilical cords can be stored for prolonged periods of time, there are currently no strict indications about which form is the most suitable for banking. Banking WJ-MSCs provides the opportunity for the autograft or allograft transplantation of MSCs in the treatment of many disorders [19,20,21]. As the human umbilical cord (UC) is regarded as “medical waste” and is usually thrown away after birth, the isolation of MSCs from UC does not cause ethical controversy. Since the UC is an easily accessible source of MSCs, many preclinical studies and clinical trials concern the umbilical cord’s Wharton’s jelly [22,23] (www.clinicaltrials.gov, accessed on 1 November 2022). Currently there are 73 active clinical trials in the world regarding MSCs, 16 of which concern MSCs derived from UC (www.clinicaltrials.gov, accessed on 1 November 2022, by using the search “active, not recruiting”). Most of the ongoing clinical trials concern central nervous system diseases (*n* = 13), brain diseases (*n* = 11), respiratory tract diseases (*n* = 13), premature birth (*n* = 10), neurodegenerative diseases (*n* = 10), and musculoskeletal diseases (*n* = 10) (www.clinicaltrials.gov, accessed on 1 November 2022). Although the use of MSCs in cell therapies seems very promising, it is important to point out that this kind of treatment is still considered “experimental”. Even though numerous clinical trials have already been completed, the obtained results are often incoherent and ambiguous. Most trials confirm the safety of MSCs therapies, but they do not deliver any results concerning the efficacy of the aforementioned treatments. This fact is associated with incoherent experimental protocols—differences in cell isolation methods, in vitro culture, storage conditions, and cell administration, which strongly influence the therapeutic secretory properties [17,24,25,26]. It seems crucial to establish standardized protocols concerning the manufacturing process of a medicinal product (that would be delivered to patients). We would like to present a proposal of a protocol selecting the most efficient isolation method of MSCs from the human UC, a favorable way of banking (as WJ-MSCs are currently banked as cell suspensions or bioptats of freshly harvested tissue), and the optimal transportation medium ensuring the best cell viability (for cells that are being delivered to patients). Concerning the optimal transport solution, in which the cells would be later administered to patients, there is no standardized or recommended medium for that purpose. As the immunomodulative properties of MSCs support the final therapeutic effect, we also focused on the soluble secretome of MSCs and WJ bioptats exposed to different conditions—different time of transportation or exposition to low temperature (−80 °C). Considering the application of WJ-MSCs for neurodegenerative diseases by intrathecal transplantation, it was also crucial for us to investigate the changes in the cell phenotype of MSCs cultured in cerebrospinal fluid.

## 2. Results

### 2.1. Isolation of MSCs from Different Regions of the Umbilical Cord (UC)

**Figure 1:**
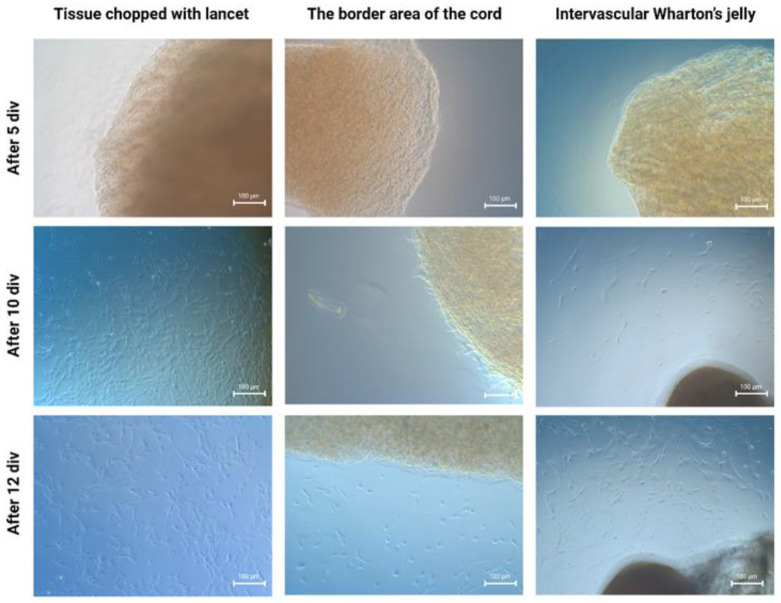
Isolation of MSCs from different regions of the UC. The rate of cell migration out of bioptats and tissue fragments was observed for 12 days (div—days in vitro) (*n* = 3). Scale bars: 100 µm.

**Figure 2:**
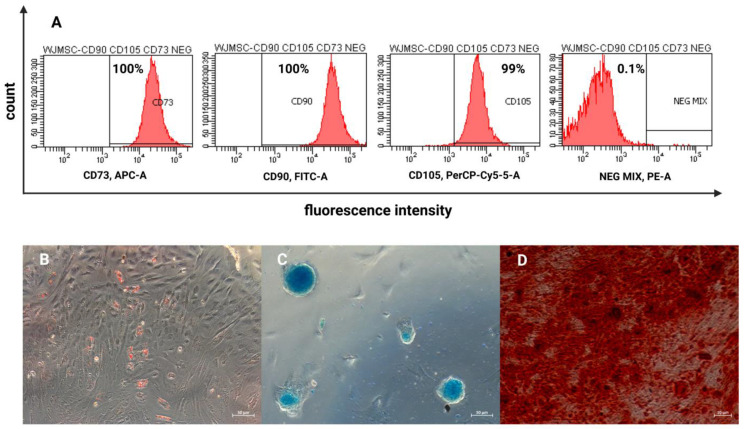
(**A**) Flow cytometry analysis. The number of cells expressing CD determines the peak in the lower-right quadrant. The latter analysis showed culture with a relatively clear expression of specific mesenchymal markers (CD73, CD90, and CD105)—more than 99% positive markers. Not more than 1% of the WJ-MSCs expressed negative markers (CD34, CD11b, CD19, CD45, and HLA-DR); (**B**) adipogenesis—positive Oil Red O staining (red fat drops), scale bar: 50 µm; (**C**) chondrogenesis—positive Alcian blue staining for the presence of cartilage glycosaminoglycans, scale bar: 50 µm; (**D**) osteogenesis—positive Alizarin Red staining, scale bar: 50 µm.

In our previous experiments, we compared the mechanical and enzymatic methods of isolating WJ-MSCs and, despite the much shorter isolation time, the mechanical method seems to result with the subpopulation of cells possessing better clonogenic and differentiation potential [26]. For this reason, in our current work, we have only compared mechanical techniques. Three different isolation methods were compared: (1) cylindrical fragments of intervascular WJ were obtained from UC slices; (2) cylindrical fragments were obtained from the border area of the cord slices; (3) the blood vessels and the epithelium were removed, and the remaining tissue was chopped into small pieces. The rate of cell migration out of the bioptats and tissue fragments was observed for 12 days. For the first 5 days no migration was observed in any of the experimental variants. After being cultured for 10 days, the first cells to migrate out into the medium were those from the tissue fragments previously chopped with a lancet (variant 3). These cells exhibited a fibroblast-like morphology, which is characteristic for MSCs. Subsequently, the intervascular Wharton’s-jelly-derived MSCs revealed their presence (variant 1), but they were less numerous than those of variant 3. At this point, no cells migrating out of the bioptats derived from the border area of the cord were observed (variant 2). After 12 days of culture, even more cells migrating out of the chopped tissue fragments and intervascular WJ bioptats were observed. At that time, cells migrating out of the bioptats derived from the border area of the cord began to appear for the first time (Figure 1). The most efficient isolation method of WJ-MSCs proved to be the one that involves cutting the whole UC with a lancet (after removing the blood vessels and the epithelium surrounding the cord). These cells had the highest migratory properties and were characterized by the highest proliferation rate. Cells derived by all the investigated methods expressed characteristic mesenchymal markers—CD73, CD90, and CD105 (Figure 2A). Isolated MSCs were able to differentiate into adipocytes—regular red fat drops were present in the cytoplasm of cells in Oil Red O staining (Figure 2B). Staining with Alcian blue and Alizarin confirmed that the MSCs differentiated into chondrocytes and osteocytes, respectively (Figure 2C,D).

### 2.2. Cell Viability in Different Transport Media in Time

**Figure 3:**
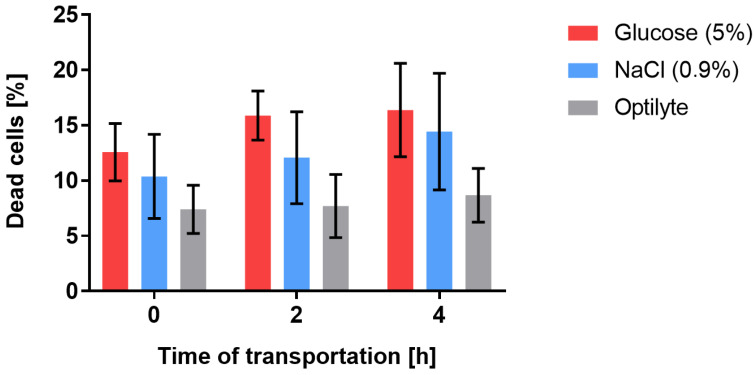
Cell viability in different transport media in time (*n* = 3). The results are presented as mean ± SD.

In order to ensure the highest viability of the WJ-MSCs destined for transplantation, a study concerning three different transportation media was conducted. The selected media, which are widely used in clinical practice, were Optilyte (multi-electrolyte solution without glucose), NaCl (0.9% solution), and glucose (5% solution). The viability of cells was examined at the beginning of the experiment and then after 2 and 4 h of simulated transportation (4 °C). The percentage of non-viable cells (stained with propidium iodide) was analyzed using FACS Canto II with FACSDiva Software (*n* = 3). The highest percentage of dead cells was detected in the 5% glucose solution (12–16%). A great number of dead cells was also detected in the 0.9% NaCl solution (10–14%). Optilyte seemed to ensure the highest viability of transported WJ-MSCs, as the percentage of non-viable cells in this medium was 7–8%. In all of the selected media, the percentage of dead cells increased with the time of transportation. When it comes to Optilyte, only slight differences in viability were observed over time (an increase by 0.3–1.2%). Regarding the 0.9% NaCl and 5% glucose, the differences in viability were higher compared to those in Optilyte. The recorded 7–12% of non-viable cells at the beginning of the experiment (in all of the media) was associated with the consequences of the cryopreserving and thawing procedures preceding the experiment. The above-mentioned procedures might have had a negative impact on the cell membrane and, thus, led to cell damage (Figure 3). According to the obtained results, the medium that ensures the highest cell viability during transportation is Optilyte. The optimal time of transportation proved to be the range of 0–2 h. The acquired results have translational potential—they provide crucial information for composing a protocol for the manufacturing and delivery of an advanced-therapy medicinal product to patients. 

### 2.3. Soluble Secretome of WJ-MSCs and WJ Bioptats

The therapeutic properties of WJ-MSCs are closely related to their secretome, which consists of many chemokines, cytokines, and growth factors. The important factor that needs to be taken into consideration when it comes to translational medicine is the form of cell banking—MSCs could be stored in the form of tissue bioptats or isolated WJ-MSCs. In this study, soluble secretomes of the WJ bioptats and isolated WJ-MSCs were compared, as the tissue bioptats (representing a heterogenous population of cells) were expected to secrete different levels of selected factors than the homogenous WJ-MSCs. Another factor that is crucial when it comes to ensuring the best quality of isolated cells is the time of transportation required for the delivery of the UCs to the laboratory. The levels of the BDNF, HGF, MCP-1 (CCL2), sICAM-1, beta-NGF, and VEGF secreted by the WJ bioptats and WJ-MSCs obtained from the UCs, which had been transported for 4, 24, and 48 h, were investigated. The term “freshly isolated”, which will be frequently used in this section, refers to WJ-bioptats that had not been cryopreserved or WJ-MSCs that had not been derived from previously cryopreserved tissue (bioptats).

#### 2.3.1. Soluble Secretome of Freshly Isolated WJ Bioptats

**Figure 4:**
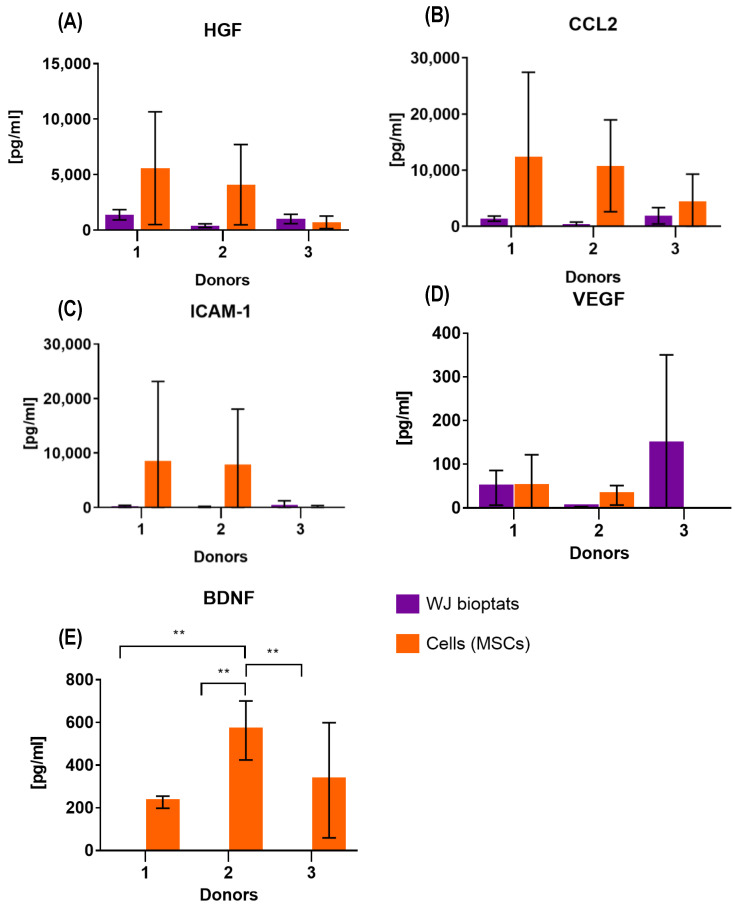
Comparison of the soluble secretomes of freshly isolated WJ bioptats and WJ-MSCs obtained from these bioptats. The results are presented as mean ± SD for each of the donors (*n* = 3), for ** < 0.01. Secretion of (**A**) HGF, (**B**) CCL2, (**C**) ICAM-1, (**D**) VEGF, and (**E**) BDNF.

The levels of the secreted factors differed between donors. The WJ bioptats obtained from all three donors secreted HGF and CCL2 (Figure 4A,B). Considering donor no. 1 (D1), the levels of secreted HGF increased with the time of transportation. The lowest levels of HGF and CCL2 were detected for the bioptats obtained from donor no. 2 (D2). The bioptats that were obtained from the UCs that had been transported for 4 h produced very low levels of ICAM-1 (which was recorded for all three donors) (Figure 4C). The WJ bioptats derived from D2 did not secrete VEGF. When it comes to the other donors, the secretion of VEGF was barely detectable, excluding that bioptats that had been derived 4 h postpartum from donor no. 3 (D3)—in this variant, the secreted levels were very high (Figure 4D). The freshly isolated tissue bioptats did not produce BDNF or beta-NGF (Figure 4E).

#### 2.3.2. Soluble Secretome of Freshly Isolated WJ-MSCs

Similar to that of the WJ bioptats, the soluble secretome of freshly isolated WJ-MSCs differed considerably between donors. Cells derived from all the UCs secreted HGF and CCL2, except the ones isolated from D1—no secretion of CCL2 was detected for the WJ-MSCs isolated after 24 h. The secretion of HGF was not detectable 4 h postpartum for D2. The lowest levels of HGF and CCL2 were noted for D3 (Figure 4A,B). Cells derived from all the UCs secreted very low levels of ICAM-1 or did not secrete it at all. The highest concentrations of ICAM-1 were noted for the WJ-MSCs obtained from D1 and D2, 4 h postpartum (Figure 4C). When it comes to VEGF, the highest concentration of this factor was noted for the cells derived from D1 4 h postpartum, while no secretion of VEGF was noted for the cells derived from D3 (Figure 4D). Unlike the WJ bioptats, the WJ-MSCs derived from all three donors secreted BDNF—the highest amounts of this factor were observed for cells isolated 4 h postpartum (Figure 4E). The WJ-MSCs did not secrete beta-NGF.

#### 2.3.3. Soluble Secretome of Cryopreserved WJ Bioptats

**Figure 5:**
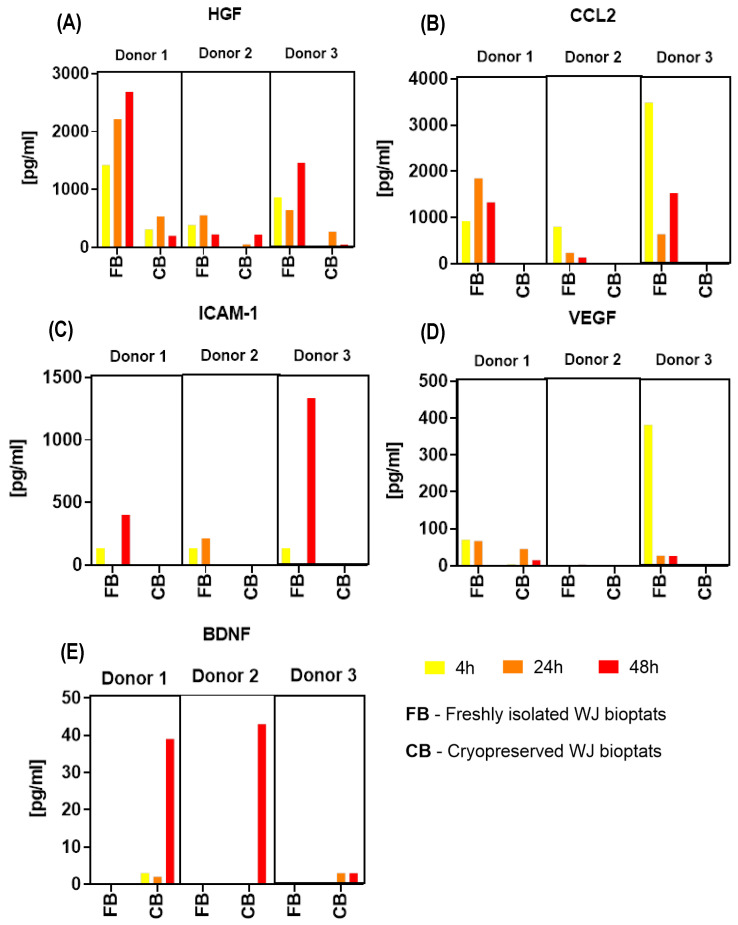
Comparison of the soluble secretomes of freshly isolated WJ bioptats and cryopreserved WJ bioptats. The results are presented as mean ± SD for each of the donors (*n* = 3). Secretion of (**A**) HGF, (**B**) CCL2, (**C**) ICAM-1, (**D**) VEGF, and (**E**) BDNF.

WJ bioptats that had been derived from D1 and D2 4 h postpartum and then cryopreserved (−80 °C) did not secrete HGF. However, they secreted large amounts of BDNF (48 h postpartum). No secretion of BDNF was detected for bioptats obtained from D2 4 and 24 h postpartum. No secretion of or very low concentrations of VEGF were detected (D2 and D3) (Figure 5). Cryopreserved WJ bioptats did not secrete beta-NGF, CCL2, or ICAM-1.

#### 2.3.4. Comparison of the Soluble Secretomes of Freshly Isolated WJ Bioptats and WJ-MSCs

After comparing the secretomes of freshly isolated bioptats and freshly isolated WJ-MSCs, it was observed that the level of secreted cytokines and chemokines was significantly higher for isolated MSCs than cultured bioptats (regardless of the time that had passed before isolation). WJ-bioptats did not secrete BDNF, while the isolated MSCs secreted high levels of this factor (Figure 4).

#### 2.3.5. Comparison of the Soluble Secretomes of Freshly Isolated WJ Bioptats and Cryopreserved WJ Bioptats

After comparing the secretomes of freshly isolated WJ bioptats and bioptats that had been cryopreserved, it was observed that the WJ bioptats that had not been exposed to a low temperature (−80 °C) secreted higher levels of HGF and CCL2 than those that had been cryopreserved. Tissue bioptats that had been exposed to a −80 °C temperature did not secrete beta-NGF, CCL2, ICAM-1, or VEGF (only in the case of one of the donors was VEGF secreted, though on very low levels); they did, however, secrete high levels of BDNF in comparison to the non-cryopreserved tissue (but only the bioptats obtained from the UCs that had been transported for 48 h). The freshly isolated bioptats did not secrete BDNF or beta-NGF (Figure 5).

### 2.4. Morphology of WJ-MSCs Cultured in CSF Obtained from Patients

**Figure 6:**
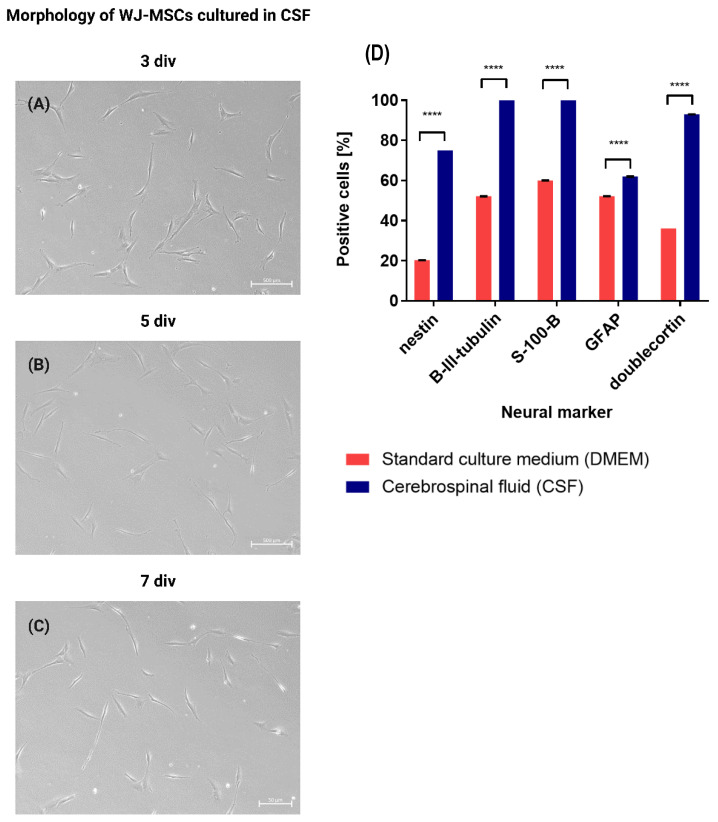
(**A**–**C**) Morphology of WJ-MSCs cultured in CSF obtained from patients. Morphology: (**A**) at 3 div; (**B**) at 5 div; (**C**) at 7 div. Scale bars: 500 µm. (**D**) Immunofluorescence staining of neural markers in WJ-MSCs. Expression of neural markers in WJ-MSCs cultured in standard culture medium (DMEM) and cerebrospinal fluid (CSF) is shown as a percentage of positive cells for each marker. The results are presented as mean ± SD, for **** < 0.0001, *n* = 9.

As WJ-MSCs are believed to exhibit therapeutic properties and can potentially be applied in the treatment of neurodegenerative diseases, it is important to investigate the fate of intrathecally transplanted cells. A study concerning changes in the cell phenotype of the WJ-MSCs exposed to CSF was conducted. The cells had been cultured in the presence of CSF for 7 days. During this time, changes in the cell morphology and proliferation rate were observed. While being cultured in the presence of CSF, the cells became elongated and formed axon-like protrusions over time (Figure 6A–C). The cells exhibited a high proliferation rate for the first 3 div, which then slowed down considerably between 3 and 5 div. The cells stopped proliferating between 5 and 7 div.

### 2.5. Expression of Neural Genes and Markers in WJ-MSCs Cultured in CSF (RT-qPCR Analysis and Immunocytochemistry)

**Figure 7:**
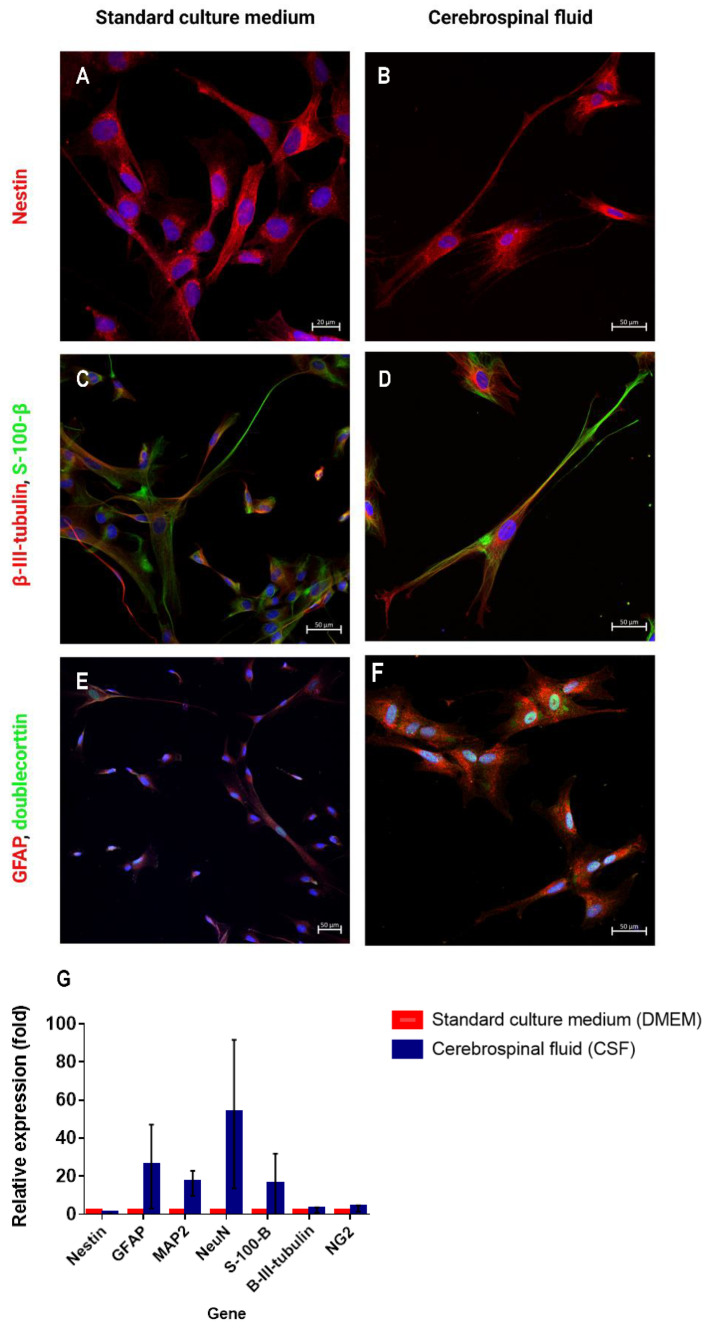
(**A**–**F**) Immunocytochemistry staining of neural markers in WJ-MSCs cultured in standard culture medium (DMEM) and cerebrospinal fluid (CSF). Scale bars: 20 µm (**A**) and 50 µm (**B**–**F**). (**G**) RT-qPCR analysis. Relative gene expression level (fold change, mean ± SD, and *n* = 9) of neural genes in WJ-MSCs cultured in standard culture medium (DMEM) and cerebrospinal fluid (CSF).

In order to investigate whether WJ-MSCs cultured in CSF undergo neural differentiation, an immunofluorescence staining was performed (WJ-MSCs cultured in standard culture medium served as the control). The cells were stained in order to detect neural markers expression such as nestin, β-III-tubulin, S-100-β, GFAP, and doublecortin. Nestin, β-III-tubulin, and doublecortin are expressed by immature neurons. S-100-β and GFAP are markers that are characteristic of astrocytes. WJ-MSCs, which had been cultured in both standard culture medium and CSF, expressed the aforementioned markers. Considerably higher expression levels of each of the analyzed markers were detected for the WJ-MSCs cultured in CSF, compared to the cells cultured in standard culture medium (Figure 6D and Figure 7A–F) Additionally, the gene expression levels for cells cultured in CSF and standard culture medium were analyzed using RT-PCR (genes coding nestin, GFAP, MAP2, NeuN, β-III-tubulin, S-100-β, and NG2). Β-actin was considered the gene of reference. The results of the RT-PCR analysis confirmed the previously obtained immunofluorescence results. The expression of most of the analyzed neural genes was considerably higher in the WJ-MSCs cultured in CSF, compared to the cells cultured in standard culture medium. However, when it comes to nestin, NG2, and β-III-tubulin, the expression level of these genes was decreased in the WJ MSCs cultured in CSF (Figure 7G). It can be assumed that in the presence of CSF, MSCs undergo neural differentiation—CSF promotes neural differentiation.

## 3. Materials and Methods

### 3.1. Isolation of MSCs from Different Regions of the Umbilical Cord (UC)

Human umbilical cords (UCs) were obtained from full-term deliveries according to the ethics committee of Warsaw Medical University, guideline KB/213/2016. UCs were transported in Optilyte (NaCl, CH_3_COONa, C_3_H_4_(OH)(COONa)_3_, CaCl_2_, KCl, MgCl_2_Na^+^, CH_3_COO^−^, Ca^2+^, and C_6_H_5_O_7_^3−^; Fresenius Cabi, Poland) to the laboratory within 2 h after birth. The 15–20 cm long UCs were cut with lancet to 2–3 mm thick slices, and

**Figure 8:**
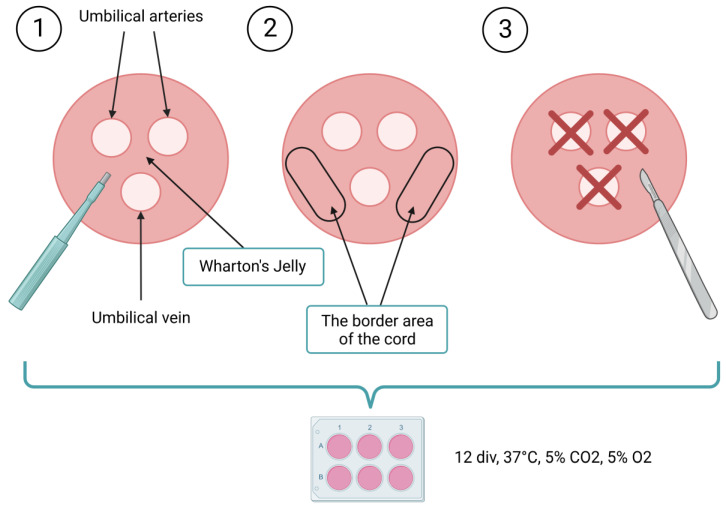
Experimental design.

- cylindrical fragments of intervascular Wharton’s jelly (WJ) were obtained from the slices using a 2 mm diameter biopsy punch (Miltex, GmbH, Viernheim, Germany)—Figure 8(1); or
- cylindrical fragments were obtained from the border area of the cord slices using a 2 mm diameter biopsy punch—Figure 8(2); or
- the blood vessels were removed using a 3 mm diameter biopsy punch, the epithelium was removed with a lancet, and the remaining tissue was chopped with a lancet into small pieces (approximately 4 mm^2^)—Figure 8(3).

The obtained bioptats and tissue fragments were then placed into 6-well culture plates and cultured in the standard cell culture medium for WJ-MSCs: DMEM (Gibco), 10% human platelet cell lysate (Macopharma, Tourcoing, France), mix of penicillin, streptomycin, amphotericin B (1:100; Gibco, Thermo Fisher Scientific, Waltham, MA, USA), 2 µg/mL heparin (Sigma-Aldrich), 37 °C temperature, 95% of humidity, 5% concentration of CO_2_, and 5% concentration of O_2_. The culture medium was replaced every 2 days for 12 div (days in vitro) (Figure 8). The migration rate of the cells out of bioptats and tissue fragments was observed to be 1, 3, 5, 7, 10, and 12 div under microscope Axio Vert.A1 (Carl Zeiss, Oberkochen, Germany).

### 3.2. Flow Cytometry Analysis

In order to analyze surface markers characteristic for MSCs, Human MSC Analysis Kit was used (Becton Dickinson, Franklin Lakes, NJ, USA). In accordance with the protocol of the manufacturer, fluorochrome-conjugated antibodies directed against APC CD73, FITC CD90, and PerCPCy_5.5 CD105 (positive markers) and PE CD34, PE CD11b, PE CD19, PE CD45, and PE HLA-DR (negative markers) were added to each sample. The samples were incubated for 30 min in the dark at room temperature. The cells were analyzed using FACSDiva software with the FACSCanto II program (Becton Dickinson, New Franklin Lakes, NJ, USA).

### 3.3. Mesodermal Lineage Differentiation

#### 3.3.1. Adipogenesis

After the cells reached a proper confluence, the standard culture medium was changed to differentiation medium from the Adipogenesis Differentiation Kit (Gibco, Thermo Fisher Scientific, Waltham, MA, USA). After 14 days of differentiation, cells were fixed with 4% PFA for 30 min and washed with PBS. After that, 60% isopropanol was added for 5 min. Staining was performed with 99% isopropanol and Oil Red O (Sigma-Aldrich, Saint Louis, MO, USA). The solution was diluted in distilled water (3:2). After removing isopropanol, the stain was added for 5 min in order to verify the positive effect of differentiation.

#### 3.3.2. Chondrogenesis

MSCs were cultured according to previously described standard conditions. After removing the culture medium, the cells were detached by accutase and centrifuged. MSCs were then seeded as a 5 µL drop/well in a 24-well plate and incubated for 60 min at 37 °C. After that, a differentiation medium was added, and the cells were cultured for 14 days. The cells were then fixed with 4% PFA. The fixed cells were then incubated with a 1% solution of Alcian blue, which detects the presence of cartilage glycosaminoglycans, (Sigma-Aldrich, Saint Louis, MO, USA) in 0.1 N HCl, for 30 min at room temperature. Excess dye was then rinsed off with 0.1 N HCl.

#### 3.3.3. Osteogenesis

After the cells reached a proper confluence, the standard culture medium was changed to differentiation medium from an Osteogenesis Differentiation Kit (Gibco, Thermo Fisher Scientific, Waltham, MA, USA). After 21 days of differentiation, cells were fixed with 4% PFA for 30 min and then washed with PBS. The cells were washed twice with distilled water and stained with 2% Alizarin Red S (Sigma-Aldrich, Saint Louis, MO, USA). In order to stain the cells, dye solution was applied for 3 min before rinsing with distilled water.

### 3.4. Evaluation of Cell Viability in Different Transport Media in Time

**Figure 9:**
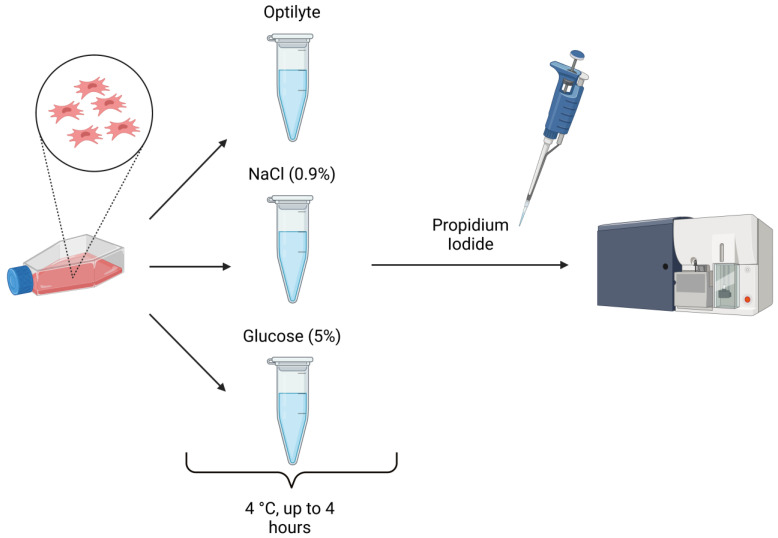
Experimental design.

Previously cryopreserved WJ-MSCs (in cryopreserving medium: DMEM, 10% human platelet cell lysate (Macopharma, Tourcoing, France), mix of penicillin, streptomycin, amphotericin B (1:100; Gibco, Thermo Fisher Scientific), 10% DMSO, −80°C) had been thawed and cultured under previously described conditions until reaching the confluence of ~80%. The cells were then harvested with Accutase Cell Detachment Solution (Beckton Dickinson) and suspended in three different transport media: Optilyte (NaCl, CH_3_COONa, C_3_H_4_(OH)(COONa)_3_, CaCl_2_, KCl, MgCl_2_Na^+^, CH_3_COO^−^, Ca^2+^, and C_6_H_5_O_7_^3−^; Fresenius Cabi, Poland), NaCl 0.9% solution (Fresenius Cabi, Poland), and glucose 5% solution (dissolved in water for injection, NaOH, and HCl to determine the pH; osmolarity—278 mOsm/L; pH: 3.5–6.5 Fresenius Cabi, Poland). The cells were then stored at 4 °C up to 4 h, mimicking the conditions of transportation of the medicinal product from the laboratory to the clinic. Cell viability was analyzed at the beginning of the experiment and then after 2 and 4 h of simulated transportation. The cells were stained with propidium iodide (according to the ThermoFisher Scientific protocol: Viability Staining Protocol for Flow Cytometry, Protocol A; ThermoFisher Scientific) and then analyzed using FACS Canto II (Beckton Dickinson, Franklin Lakes, NJ, USA) with FACSDiva Software (Beckton Dickinson) (Figure 9).

### 3.5. Soluble Secretome of WJ-MSCs and WJ Bioptats

**Figure 10:**
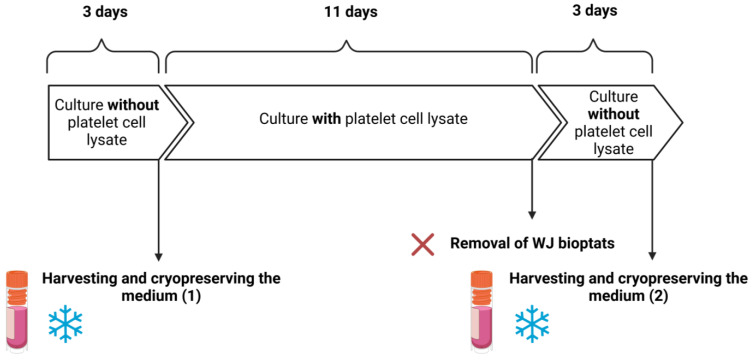
Experimental design.

Human umbilical cords (UCs) were obtained from full-term deliveries (*n* = 3) according to the ethics committee of Warsaw Medical University, guideline KB/213/2016. The 15–20 cm long UCs were cut into 3 fragments of equal length. Each fragment was kept at 4 °C in Optilyte for either 4, 24, or 48 h. After that time, UC fragments were cut with lancet to 2–3 mm thick slices, and bioptats of WJ were obtained from the slices using a 2 mm diameter biopsy punch (Miltex, GmbH, Viernheim, Germany). Then, the freshly isolated bioptats were either: (1) placed on a 6-well culture dish and cultured according to previously described conditions for the first 3 days, except the culture medium did not contain human platelet cell lysate (DMEM (Gibco), mix of penicillin, streptomycin, amphotericin B (1:100; Gibco, Thermo Fisher Scientific), 2 µg/mL heparin (Sigma-Aldrich), 37 °C, 95% of humidity, 5% CO_2_, and 5% O_2_), (2) or they were cryopreserved for duration of 3 months (in cryopreserving medium: DMEM, 10% human platelet cell lysate (Macopharma, Tourcoing, France), mix of penicillin, streptomycin, amphotericin B (1:100; Gibco, Thermo Fisher Scientific), 10% DMSO, and −80 °C). After 3 days, the culture medium was harvested and stored at −80 °C for future Luminex Assay analysis. The bioptats were then cultured in standard culture medium (containing 10% human platelet cell lysate) until 14 div. After 14 div, when a great number of WJ-MSCs migrating out of the bioptats was observed, the bioptats and culture medium were removed. Isolated WJ-MSCs were then cultured for 3 additional days in a medium lacking 10% human platelet cell lysate. After that time, the medium was harvested and stored at −80 °C for future Luminex Assay analysis, same as before. The same procedure was repeated for WJ bioptats that had been cryopreserved, although no cells were obtained (Figure 10). The withdrawal of human platelet cell lysate was necessary for receiving reliable results concerning WJ-MSCs soluble secretome, as human platelet cell lysate contains a great number of different factors itself. On the day of analysis, the cryopreserved media were thawed and prepared, in accordance with the instructions and attached protocol of the kit (Luminex Assay, R&D Systems, Minneapolis, MN, USA). Levels of HGF, BDNF, sICAM-1, MCP-1 (CCL2), beta-NGF, VEGF secreted by cells, and bioptats into the media were analyzed on Luminex 200 Bio-Rad.

### 3.6. Characteristics of WJ-MSCs Cultured in Cerebrospinal Fluid Obtained from Patients

Cerebrospinal fluid was obtained from patients according to the ethics committee of Warsaw Medical University, guideline AKBE/242/2022.

#### 3.6.1. Morphology

WJ-MSCs were seeded on 6-well plate in the amount of 4 × 10^3^/cm^2^. Cells were cultured in the presence of cerebrospinal fluid (CSF) obtained from patients for 7 days (37 °C, 95% of humidity, 5% CO_2_, and 5% O_2_). Cell morphology was examined at 3, 5, and 7 div under microscope Axio Vert.A1 (Carl Zeiss, Oberkochen, Germany).

#### 3.6.2. Expression of Neural Genes by Real-Time Quantitative Polymerase Chain Reaction (RT-qPCR)

WJ-MSCs were seeded on T25 culture flasks in the amount of 4 × 10^3^/cm^2^ and cultured in WJ-MSCs standard medium until reaching a confluence of ~40%. At this point, the medium was replaced with cerebrospinal fluid, and the cells were cultured for 3 additional days. Total RNA was isolated from the cell population using the Total RNA Mini Plus Concentrator (A&A Biotechnology), in accordance with the protocols of the manufacturer. RNA was eluted with 20 µL of RNase-free H2O (Sigma Aldrich). The quantity and quality of RNA were assessed using a NanoDrop 2000 spectrophotometer (Thermo Scientific). A complementary strand of DNA (cDNA) from RNA was generated using a High-Capacity RNA-to-cDNA™ Kit (Applied Biosystems, Thermo Fischer Scientific, Waltham, MA, USA), in accordance with the attached protocol. Real-time quantitative polymerase chain reaction (RT-qPCR) was performed using SYBR green Master Mix (Applied Biosystems) and specific primers (Table 1) with the 7500 Real Time PCR System (Applied Biosystems) (Table 2). Β-actin was considered the gene of reference.

#### 3.6.3. Immunocytochemistry

Immunocytochemistry was performed to detect the expression of neural genes in isolated WJ-MSCs’ population cultured in the presence of CSF. The cells were washed with phosphate buffer saline (PBS) solution (PBS; Sigma-Aldrich, Saint Louis, MO, USA) and fixed in 4% PFA for 15 min. Obtained samples were incubated with 0.2% Triton X-100 (Sigma-Aldrich), 1% BSA (Sigma-Aldrich), and 10% GS (Sigma-Aldrich) for 1 h at room temperature. Next, primary antibodies were applied for 24 h, and the samples were kept at 4 °C (Table 3). On the following day, the cells were washed with PBS and then incubated with secondary antibodies conjugated with fluorochrome for 1 h (Table 4). Cell nuclei were stained with DAPI (Invitrogen, Waltham, MA, USA). The analysis was performed using confocal microscope Zeiss LSM780 (Carl Zeiss).

## 4. Discussion

### 4.1. Most Efficient Isolation Method of MSCs from Human Umbilical Cord

Evaluating the most efficient isolation method of MSCs from the human UC is crucial when considering future clinical applications. In our previous work, we investigated two different isolation procedures: WJ-MSCs were isolated by either mechanical- or enzymatic-based procedures. Despite the initially higher proliferation rates observed in the first passages of the WJ-MSCs derived enzymatically, the further passaging of both types of differently isolated WJ-MSCs’ cultures confirmed that only the mechanical method can provide steadily proliferating cells for a longer time period. Moreover, WJ-MSCs isolated enzymatically lacked the capability to differentiate into multiple lineages, together with a drastic decrease in cell CFU (colony forming unit)-formation efficiency when compared to cells derived mechanically [26]. Hendijani et al. compared two different MSCs’ isolation methods for the human UC: (1) the authors obtained bioptats from intervascular WJ; (2) the authors made incisions on 1.5 cm-long fragments of the UC, in order to expose a wider area of tissue to contact with the culture medium (the blood vessels were not removed). After 24 h in vitro, the first cell migration was observed. The cells from both variants proliferated for the next 15 days, although the cells derived from the WJ bioptats formed denser colonies than those obtained from the incised UC fragments [27]. Subramanian et al. compared cell isolation efficiency from WJ, intervascular WJ, and UC epithelium. According to the results, cells derived from WJ demonstrated a higher proliferation rate and were more viable [28]. On the other hand, Ishige et al. proved that cells obtained from the UC’s arterial wall proliferated the fastest in culture, when compared to cells isolated from WJ [29]. We focused on the umbilical cord matrix to obtain a more homogeneous population, depleted of epithelial cord lining stem cells, perivascular cells, endothelial precursors, and smooth muscle fibers. It can be assumed that the method involving cutting the whole UC with a lancet (after removing the blood vessels and the epithelium surrounding the cord) enables better cell migration, as larger tissue fragments interact with the base of the culture plate.

### 4.2. Most Optimal Culture and Long-Term Storage Conditions

Previously, we have shown that cell cultivation under low oxygen (5%), which mimics physiological normoxia, ensures the best cell quality. Cell cultivation under 21% oxygen must be considered as non-physiological, as it is said to induce oxidative stress in cultured cells by the increased production of reactive oxygen species (ROS). WJ-MSCs cultured under 5% O_2_ displayed a higher proliferation rate than those cultured under 21% O_2_. They were also characterized by an unchanged plasticity for differentiation into the three germ layers and a stable, correct karyotype [5,26]. When it comes to long-term cryostorage, our results show that it is best to cryopreserve the isolated cells in a suspension, not as bioptats, as no WJ-MSCs were observed to migrate out of WJ bioptats after thawing. Consistent with our results, Theofanis et al. stated that no MSCs’ cultures were obtained from WJ samples cryopreserved for up to 6 months in liquid nitrogen, despite the cryoprotection from 10% dimethylsulfoxide (DMSO) and 5% glycerol [30]. It was also confirmed that the therapeutic potential of MSCs reduces significantly during cryostorage [31]. In this paper, we show that the secretory properties of MSCs decrease after exposure to low temperatures.

### 4.3. Most Optimal Transport Medium for WJ-MSCs

Optimizing cell transportation conditions, which ensure the highest cell viability, is crucial when it comes to achieving a therapeutic effect. It is important to choose the most optimal medium for cell transportation that not only is the best for cell viability but also can be administered later to patients. The chosen media must comply with the EMA (European Medicines Agency) guideline and must be specifically dedicated for clinical use. Most studies concern the use of PBS for cell transportation, although it does not comply with the EMA guideline, as PBS cannot be administered intrathecally. In this study, only media that can be used in clinical practice were investigated (Optilyte, 5% glucose, and 0.9% NaCl). Iftimia-Mander et al. stored the UC for 24, 72, and 120 h after birth, mimicking the tissue transportation conditions. The authors kept in mind that the time of transportation might increase due to logistic difficulties, etc., although no significant differences depending on the amount of time that had passed were observed. Cell isolation proved to be slightly more efficient, when it comes to UCs that had been transported for 72 h [32]. Celikkan et al. transported the UCs in L-15 medium 15 (Leibovitz; Sigma; L4386) for 12–24 h. After cell isolation, the cell viability in two different media was analyzed: PBS and Ringer’s solution. Ringer’s solution ensured higher cell viability compared to PBS—93% after 6 h of storage. These cells also proliferated faster in culture [33]. On the other hand, Petrenko et al. investigated cell viability after being stored for 24, 48, and 72 h in BTS (Buffered Trehalose Solution), Ringer’s solution, Plasma-Lyte 148 (Baxter Health-care Ltd., Deerfield, IL, USA), and HypoThermosol FRS (HTS-FRS, BioLife Solutions Inc., Bothell, WA, USA). Regardless of the medium used, the authors described the same dependence: the percentage of viable cells was the highest for the cells being stored up to 24 h—after that time the number of viable cells gradually decreased. The highest viability was observed for BTS and HypoThermosol FRS. The lowest viability was observed for Ringer’s solution [34]. In our study, the viability did not drop below 84% in any of the media (considering that the time of transportation was not more than 4 h). Similar to other reported studies, a decrease in cell viability was observed in each of the media during transportation. Unfortunately, no reference data considering chosen media were found among published papers.

After arriving at the clinic, cells can be administered intravenously, intrathecally, intracerebrally, or intranasally—depending on the disease that needs to be treated. For neurological diseases, the intrathecal injection seems to be most promising, as the circulating cerebrospinal fluid helps to distribute the injected cells and their products throughout the subarachnoid space. Another advantage is that lumbar puncture is a low- risk medical intervention [25]. When it comes to heart diseases, MSCs can be administered intravenously—this method proved to be safe in a group of patients with stable heart failure [35]. The frequency of cell application is also an important factor that plays a critical role regarding the therapeutic effect. Usually, a repetitive therapy instead of one single application is needed. Depending on the disease treated, it takes about 2–5 doses of MSCs for patients to show clinical improvement, although the therapeutic outcome varies between patients and their condition [25].

### 4.4. Soluble Secretome and Therapeutic Properties of WJ-MSCs

MSCs exhibit immunomodulatory and neuroprotective features. The effective use of MSCs in cell therapy is mostly based on the beneficial effect of secreted growth factors, chemokines, and anti-inflammatory cytokines—MSCs possess essential protective features and promote regeneration. Understanding MSCs’ soluble secretome is crucial when it comes to applying stem cell therapy in clinical treatment. WJ bioptats and isolated MSCs both secreted HGF, BDNF, ICAM-1, MCP-1, NGF, and VEGF, which is consistent with the results obtained by Teixeira et al.—although the level of secreted factors differed depending on the donor [36]. The obtained results indicate that WJ-MSCs secrete higher levels of analyzed factors than the heterogenous cell population of the UC (this population not only consists of MSCs but also of epithelial and endothelial cells). This also confirms the advantage of the mechanical cell isolation technique over the enzymatic method, as the mechanical method allows for a homogenous cell culture. The advantage of this method was also described by Lech et al. [26]. It can be assumed that with a longer time for UC transportation, the therapeutic properties of the MSCs inhabiting the tissue decrease—the level of secreted factors by WJ-MSCs and WJ bioptats decreases over time. Cryopreservation has a negative impact on the soluble secretome of WJ bioptats. However, no reference data considering this issue were found among published papers. Tomecka et al. demonstrated that WJ-MSCs secrete IFN-γ, IL-4, IL-6, HGF, TGF-β, and PGE-2. The secretion of BDNF, TNF-α, IL-10, IL-17, and VEGF was detected on a very low level [17]. Salwierak-Głośna et al. observed high levels of IL-6, IL-8, IP-10, and MCP-1 in WJ-MSCs’ secretome. The authors observed the secretion of IL-1, TNF-α, and IFN-γ (when it comes to proinflammatory cytokines) as well as IL-1ra, IL-4, IL-10, and IL13 (the cytokines involved in the anti-inflammatory response) [37]. Teixeira et al. confirmed, with the use of the Luminex method, that MSCs secrete factors such as VEGF, beta-NGF, BDNF, IL-6, and GDNF [36]. VEGF and IL-6 were secreted at a high level, while the secretion of beta-NGF, BDNF, and GDNF was low. Differences in the results of various studies focused on the secretion of beta-NGF might be related to the sensitivity level of the particular methods used. With the use of a Bio-Plex Pro Human Cytokine Group I Assay Eotaxin, Romanov et al. [38] detected very high levels of MCP-1 (CCL2) and HGF secreted by WJ-MSCs. Similar to other authors, Eleuteri et al. [39] confirmed the ability of MSCs to secrete CCL2, VEGF, and HGF. Unfortunately, no studies considering the soluble secretome of WJ bioptats were found. In this paper, it was shown that the soluble secretome of WJ-MSCs displays large inter-individual variability, despite the use of the same protocol. The fact that the secretion level of different factors varies significantly between patients needs to be taken into account when considering personalized cellular therapies. MSCs can modify the functions of the microenvironment not only by their soluble secretome but also by the release of extracellular vesicles (EVs). We can distinguish three main types of EVs: exosomes, microvesicles, and apoptotic bodies. Exosomes, being the smallest in size, contain many different factors and can have a great therapeutic potential as MSCs themselves. They are involved in intercellular communication via the transfer of numerous membrane receptors, proteins, lipids, RNA, and miRNA between cells. Currently there are many ongoing experiments regarding MSC-derived EVs, which could be applied in cellular-free therapies in the future [40,41,42,43,44].

### 4.5. WJ-MSCs Cultured in CSF Change Their Phenotype and Undergo Neural Differentiation

In order to understand the fate of MSCs administered intrathecally, it is important to investigate the changes of the cell phenotype in the presence of cerebrospinal fluid. Ge et al. [45] cultured MSCs obtained from umbilical cord blood and bone marrow in the presence of culture medium with a small addition of CSF (10 µL CSF for every 2 mL of DMEM, without platelet lysate). The authors described changes in the cell morphology after 3 days of in vitro culture: the cells became irregular, and many were triangle-shaped. After 7 days of culture, the cells formed characteristic, axon-like protrusions. After investigating the expression of neural markers (GFAP and β-III-tubulin), it was observed that cells cultured in standard culture medium did not express GFAP and β-III-tubulin. However, the addition of CSF to the medium triggered the expression of these markers. A different approach was described by Farivar et al. [46] The authors analyzed the expression of MAP2, nestin, and GFAP in WJ-MSCs cultured in standard culture medium with the addition of 100 µL of CSF. WJ-MScs cultured in the standard medium expressed low levels of nestin and did not express GFAP and MAP2. Seven days after the addition of CSF to the standard culture medium, the cells started to express all of the aforementioned markers. Ye et al. [47] cultured MSCs derived from bone marrow in standard culture medium (IMDM) for 7 days—every day they added 10 µL of CSF. After 4 div, the authors observed changes in the cell morphology—the cells became triangular and irregular in shape. After 7 div, the cells became elongated, and many axon-like protrusions could be observed. Similar to Ge et al. [45], the authors investigated the expression of GFAP and β-III-tubulin. GFAP+ and β-III-tubulin+ cells appeared in the culture that was supplemented with CSF. In our study, it was proven that WJ-MSCs cultured in CSF expressed higher levels of neural genes than those cultured in standard culture medium. The unique interaction of MSCs with the CSF that promotes neural differentiation might be related to the presence of many growth factors such as bFGF and EGF [48]. It has been proven, during regeneration after brain injury, the levels of the IGFs, FGFs, NGF, TGF-β, GDNF, BDNF, VEGF, and neurotrophins in CSF significantly increase. These factors have neuroprotective properties, stimulate growth, and improve the survival rate of neurons as well as the proliferation of neural stem cells [49]. Considering the morphological changes in culture and the results of immunocytochemistry staining and RT-PCR, it can be assumed that in the presence of CSF, mesenchymal stem/stromal cells undergo neural differentiation. The obtained results are of great importance when it comes to clinical applications, as they tell us more about the fate of the WJ-MSCs transplanted into patients’ CSF and their therapeutic properties.

## 5. Conclusions

In our opinion, in order to obtain WJ-MSCs with the best therapeutic properties, it is best to

- isolate the cells using the mechanical method, which involves cutting the whole UC with a lancet (after removing the blood vessels and the epithelium surrounding the cord);
- transport the cells in Optilyte (a multi-electrolyte medium), as it proved to be the medium that ensures the highest cell viability during transportation, with the optimal time of transportation being 0–2 h; and
- cryopreserve the isolated cells and not the tissue bioptats (concerning long term stem cell-banking), as it was shown that WJ-MSCs secrete higher levels of immunomodulatory, angiogenic, and neuroprotective factors than whole WJ bioptats, and we can also conclude that low temperatures cause a decrease in the soluble secretome of WJ bioptats.

## Figures and Tables

**Table 1:** List of primers used for real-time quantitative polymerase chain reaction (RT-qPCR).

Gene Name	Specific Primers
*nestin*	F: GGGAAGAGGTGATGGAACCA R: AAGCCCTGAACCCTCTTTGC
*GFAP*	F: CCGACAGCAGGTCCATGT R: GTTGCTGGACGCCATTG
*MAP2*	F: TTGGTGCCGAGTGAGAAGA R: GTCTGGCAGTGGTTGGTTAA
*RBFOX3* (NeuN)	F: ACTTACGGAGCGGTCGTGTATC R: ATGGTGTGATGGTACGGGTCGG
*TUBB3* (β-III-tubulin)	F: GGAAGAGGGCGAGATGTACG R: GGGTTTAGACACTGCTGGCT
*S-100-β*	F: AGCGCTCCTGGAAAAAGCAA R: TTGAATCGCATGGGTCACGG
*NG2*	F: GTCTACACCATCGAGCAGCC R: TGTGTGAGAACAGCACGAGC
*ACTB* (β-actin)	F: CATGTACGTTGCTATCCAGGC R: CTCCTTAATGTCACGCACGAT

**Table 2:** List of reaction components used for real-time quantitative polymerase chain reaction (RT-qPCR).

Reaction Components	Concentration
cDNA	10 ng (2 µL)
RT HS-PCR Mix SYBR (A&A Biotechnology)	7.5 µL
Specific primers (5′ i 3′)	0.25 µM/µL (0.075 µL)
RNase-free water	5.425 µL

**Table 3:** List of primary antibodies used for immunocytochemistry.

Antigen	Source	Isotype	Dilution	Company
Nestin	Mouse monoclonal	IgG1	1:500	Merck
β-III-tubulin	Mouse monoclonal	IgG2b	1:400	Sigma
S-100-β	Rabbit polyclonal	Polyclonal	1:100	abcam
GFAP	Mouse monoclonal	IgG1	1:1000	Biolegend
Doublecortin	Rabbit polyclonal	Polyclonal	1:800	CellSignaling

**Table 4:** List of secondary antibodies used for immunocytochemistry.

Antigen	Antibody	Dilution	Fluorochrome (ThermoFisher Scientific)
Nestin	Anti-mouse	1:500	Alexa Fluor 546
β-III-tubulin	Anti-mouse	1:400	Alexa Fluor 546
S-100-β	Goat, anti-rabbit	1:100	Alexa Fluor 488
GFAP	Anti-mouse	1:1000	Alexa Fluor 546
Doublecortin	Goat, anti-rabbit	1:800	Alexa Fluor 488

## Data Availability

The data presented in this study are available on request from the corresponding author.
